# Postoperative Survival Analysis of Elective Colorectal Cancer Surgery with Liver Cirrhosis: A Propensity-Matched Study

**DOI:** 10.3390/curroncol33010029

**Published:** 2026-01-05

**Authors:** Tsung-Jung Tsai, Kai-Jyun Syu, Xuan-Yuan Huang, Yu Shih Liu, Chang-Wei Chen, Yu-Yao Chang, Yen-Hang Wu, Tsung Chuang

**Affiliations:** Division of Colon and Rectal Surgery, Department of Surgery, Changhua Christian Hospital, Changhua 500209, Taiwan; tcr0929@gmail.com (T.-J.T.); 1528495@cch.org.tw (K.-J.S.); 107431@cch.org.tw (X.-Y.H.); 184403@cch.org.tw (Y.S.L.); 182455@cch.org.tw (C.-W.C.); 185268@cch.org.tw (Y.-H.W.)

**Keywords:** liver cirrhosis, colorectal cancer, elective surgery, survival, hypoalbuminemia, MELD-Na

## Abstract

**Background:** Liver cirrhosis increases perioperative risk in colorectal cancer surgery, yet data on long-term outcomes remain limited. In this study, we evaluated postoperative morbidity, mortality, and survival in cirrhotic patients. **Methods:** In this single-center retrospective cohort, 53 cirrhotic patients undergoing elective colectomy or proctectomy (2011–2022) were propensity score-matched 1:1 with non-cirrhotic controls. Perioperative variables, complications, and survival were analyzed. Subgroup analyses were performed for right hemicolectomy and non-right hemicolectomy procedures. Kaplan–Meier and logistic regression analyses were implemented to assess outcomes and risk factors. **Results:** Cirrhotic patients had higher preoperative MELD-Na scores and lower albumin and hemoglobin levels. They experienced greater blood loss, longer operative times, more ICU admissions, and higher rates of major complications (18.9% vs. 3.8%, *p* = 0.01). Mortality was higher at in-hospital (7.5% vs. 0%), 3-month (9.4% vs. 0%), and 60-month (66% vs. 28.3%) intervals, and these patients’ overall survival was shorter (70.7 vs. 116.8 months, *p* < 0.001). The subgroup analysis showed that the adverse impact of cirrhosis persisted for both right hemicolectomy and non-right hemicolectomy procedures, with significantly worse long-term survival in cirrhotic patients. Postoperative complications after right hemicolectomy did not differ significantly between groups. Among cirrhotic patients, Child–Turcotte–Pugh class B predicted worse survival than class A (40.1 vs. 84.8 months, *p* = 0.006). Preoperative hypoalbuminemia (<3.5 g/dL) independently predicted long-term mortality (HR = 3.93). **Conclusions:** Elective colorectal surgery in patients with cirrhosis is associated with increased perioperative complications and significantly reduced long-term survival. However, postoperative outcomes after right hemicolectomy in cirrhotic patients were comparable to those of non-cirrhotic patients, despite their persistently poorer long-term survival. Optimization of nutritional status and careful preoperative assessment of hepatic reserve are essential to improving outcomes in this high-risk population.

## Simple Summary

Patients with liver cirrhosis are at increased risk of multiple complications, most notably postoperative hepatic decompensation and renal impairment. Due to the increased surgical risk associated with the condition, operative interventions are undertaken less frequently in this population, resulting in limited evidence regarding short- and long-term survival and accurate risk assessment across diverse clinical backgrounds and comorbidities. In this study, we evaluated the clinical outcomes of 53 patients with liver cirrhosis compared with non-cirrhotic controls using a propensity score-matched analysis. Our findings demonstrated that liver cirrhosis was independently associated with reduced postoperative survival, even after stratification via the Child–Turcotte–Pugh classification. Moreover, preoperative serum albumin emerged as a critical prognostic factor influencing overall survival.

## 1. Introduction

Cirrhosis is a common and serious global health problem resulting from chronic liver inflammation that progresses from a compensated to a decompensated state. As the disease advances, patients develop complications such as portal hypertension, hepatic dysfunction, renal impairment, and ascites [1]. For patients with liver cirrhosis, surgery is challenging due to thrombocytopenia related to perioperative bleeding, which makes surgeons reluctant to operate. Postoperative hepatic decompensation and renal impairment are also causes for concern. Cirrhosis is considered a risk factor for morbidity and mortality after colorectal surgery [2].

Surgical stress may further exacerbate hepatic injury; thus, accurate risk stratification is essential. Several scoring systems, such as the Child–Turcotte–Pugh (CTP) classification, the model for end-stage liver disease-sodium (MELD-Na), the Mayo Risk Score, and the VOCAL-Penn Cirrhosis Surgical Risk Score, have been proposed to assess disease severity, predict postoperative decompensation, and estimate mortality risk [3]. However, these tools are not specifically tailored to colorectal procedures and may not fully capture the complexity of this patient population.

Colorectal cancer patients with underlying cirrhosis experience significantly worse survival than non-cirrhotic patients [4]. Patients with liver cirrhosis are often excluded from cancer trials, meaning that there is little evidence or clinical data to help delineate the benefits of surgery or other treatments. Consequently, these patients remain understudied, and clinicians often struggle to weigh oncologic benefit against operative risk.

Additionally, colorectal surgical procedures carry different levels of physiologic stress. Right hemicolectomy is generally considered less complex and has a lower risk of anastomotic complications compared with anterior or low anterior resection. It is therefore plausible that cirrhotic patients may tolerate certain procedures better than others, particularly right-sided colectomy, which is typically associated with safer anastomoses and fewer postoperative complications.

In light of these factors, in this study, we aimed to retrospectively evaluate postoperative morbidity and mortality in cirrhotic patients undergoing elective colorectal surgery. A secondary objective was to determine potential risk factors that influence survival in these patients.

## 2. Materials and Methods

### 2.1. Study Population

This single-center retrospective cohort study was conducted in a tertiary medical center and approved by the institutional review board of our center (submission ID: 220619). The requirement for written informed consent was waived because of the retrospective nature of this study. The electronic medical records of patients undergoing colectomy, including open and laparoscopic procedures, between January 2011 and July 2022 were reviewed and analyzed. The inclusion criteria included (1) adult patients aged > 18 years, (2) who underwent colectomy or proctectomy, and (3) had a diagnosis of liver cirrhosis on their electronic record. Patients with familial adenomatous polyposis or hereditary non-polyposis colorectal cancer were excluded.

### 2.2. Study Design

A total of 2988 patients were enrolled, of whom 53 had liver cirrhosis. Patients underwent 1:1 propensity matching according to age, cancer location, BMI, and tumor stage. They were then divided into two groups: a cirrhosis group and a control group. The patients’ body mass indexes and albumin, hemoglobin, platelet, and carcinoembryonic antigen levels were determined prior to surgery. We calculated the preoperative model for end-stage liver disease score that includes (MELD-Na) in patients with liver cirrhosis and determined the renal function score, which included a score for the modification of diet in renal disease (MDRD). Any other underlying conditions were recorded.

### 2.3. Surgery and Follow-Up

In terms of surgical management for colorectal cancer, all enrolled patients underwent high ligation of the feeding vessels. Rectal cancer patients received total mesorectal excision. Histopathological findings were reviewed, including the presence of lymphatic invasion, venous invasion, perineural invasion, tumor diameter, and pathological difference.

Operative variables such as the duration of surgery, intraoperative blood loss, intraoperative transfusion requirement, postoperative intensive care unit (ICU) admission, and length of hospital stay were recorded. Postoperative complications were classified according to the Clavien–Dindo classification system, with major complications defined as those requiring surgical, endoscopic, or radiological intervention (Clavien–Dindo grade ≥ III).

Mortality was defined according to the timing after surgery. In-hospital mortality referred to any death that occurred during hospitalization; short-term mortality was defined as death within 3 months postoperatively; and long-term mortality was classified as death within 60 months after surgery. All patients underwent follow-up every 3 months for the first 2 years and every 6 months thereafter for a total of 5 years.

### 2.4. Subgroup Analysis: Right vs. Non-Right Hemicolectomy

A subgroup analysis was performed to compare mortality outcomes between patients with and without cirrhosis who underwent colorectal surgery. Patients were stratified according to the type of surgical procedure: right hemicolectomy and non-right hemicolectomy. All analyses were conducted separately for the right hemicolectomy and non-right hemicolectomy groups to assess whether the impact of cirrhosis on postoperative mortality differed by surgical procedure.

## 3. Results

A total of 106 patients who underwent colorectal surgery were included in this study, of whom 53 had liver cirrhosis. The remainder were cirrhosis-free. The demographic, perioperative, clinicopathological, and treatment characteristics of the study population are summarized in Table 1.

There was a higher proportion of males and a greater prevalence of coronary artery disease and diabetes mellitus in the cirrhosis group compared to the group without cirrhosis. No differences were observed in the prevalence of end-stage renal disease, heart failure, stroke, chronic obstructive pulmonary disease, or history of abdominal operation. Preoperatively, the cirrhotic group demonstrated a significantly higher MELD-Na score (12 vs. 9, *p* < 0.001), lower serum albumin levels (3.3 g/dL vs. 3.5 g/dL, *p* = 0.05), and lower hemoglobin levels (11.1 g/dL vs. 12.1 g/dL, *p* = 0.03).

Intraoperatively, patients with cirrhosis experienced greater blood loss and longer operative times. They also required more frequent perioperative blood transfusions and postoperative intensive care unit (ICU) admission. No significant differences were observed between the two groups regarding pathological characteristics, including tumor size, stage, histological differentiation, vascular invasion, lymphatic invasion, and perineural invasion.

**Figure 1:**
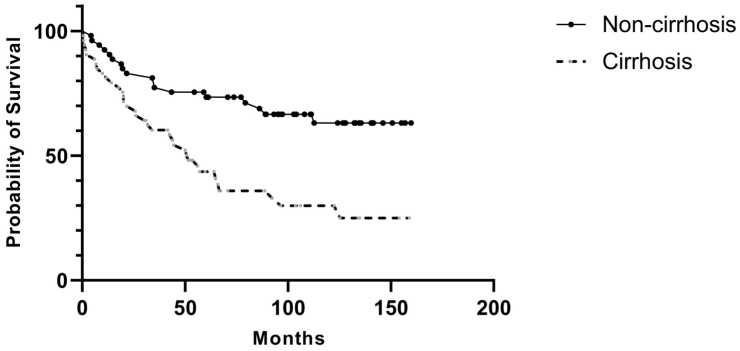
Postoperative overall survival in patients with and without liver cirrhosis.

Postoperatively, major complications were significantly more common in the cirrhosis group (18.9% vs. 3.8%, *p* = 0.01), particularly intra-abdominal abscesses (9.4% vs. 0%, *p* = 0.02) and surgical site infection (15.1% vs. 1.9%, *p* = 0.02). The cirrhosis group also had significantly longer hospital stays (18 days vs. 10 days, *p* < 0.001) and higher mortality across all timeframes, including in-hospital mortality (7.5% vs. 0%, *p* = 0.04), 3-month mortality (9.4% vs. 0%, *p* = 0.02), and 60-month mortality (66% vs. 28.3%, *p* < 0.001). The overall survival duration was substantially shorter in cirrhotic patients (70.7 months vs. 116.8 months, *p* < 0.001) (Figure 1). Most mortality was related to cirrhosis.

**Subgroup Analysis by cirrhosis and non-cirrhosis patients in right and non-right hemicolectomy groups.**

Among patients undergoing right hemicolectomy, all mortality indices were higher in the cirrhosis group. Long-term mortality was significantly increased (76.2% vs. 35.3%, *p* = 0.01), while higher in-hospital mortality (14.3% vs. 0%, *p* = 0.10) and short-term mortality (19.0% vs. 0%, *p* = 0.06) did not reach statistical significance.

For patients undergoing non-right hemicolectomy procedures, long-term mortality remained significantly elevated in the cirrhosis cohort (59.4% vs. 40.6%, *p* < 0.01), whereas differences in in-hospital and short-term mortality were not statistically significant.

**Perioperative and postoperative outcomes between cirrhosis and non-cirrhosis patients in right and non-right hemicolectomy groups (Table 2).**

In the right hemicolectomy subgroup, the operative time was similar between cirrhotic and non-cirrhotic patients (202 ± 81 vs. 167 ± 63 min, *p* = 0.16). Cirrhotic patients showed a trend toward increased perioperative transfusion (19% vs. 0%, *p* = 0.06) and postoperative ICU admission (28.6% vs. 11.8%, *p* = 0.21). The overall complication rate was higher in patients with cirrhosis (38.1% vs. 23.5%, *p* = 0.34). Major complications (Clavien–Dindo ≥ III) were also more common (23.8% vs. 5.9%, *p* = 0.13) without statistical significance, whereas minor complications and specific postoperative morbidities, including intra-abdominal abscess, ileus, surgical site infection, and anastomotic leakage, did not differ significantly. Cirrhotic patients also tended to have longer hospital stays (18 ± 12 vs. 12 ± 7 days, *p* = 0.06).

In the non-right hemicolectomy subgroup, cirrhotic patients had longer operative times (219 ± 81 vs. 187 ± 42 min, *p* = 0.09) and significantly higher rates of perioperative transfusion (21.9% vs. 2.8%, *p* = 0.02) and postoperative ICU admission (21.9% vs. 0%, *p* < 0.001). While the overall complication rate showed no significant difference (28.1% vs. 16.7%, *p* = 0.26), cirrhotic patients were more likely to experience major complications (15.6% vs. 2.8%, *p* = 0.06). Surgical site infection was also significantly more common among cirrhotic patients (12.5% vs. 0%, *p* = 0.03). Other complication rates, including intra-abdominal abscess, ileus, and anastomotic leakage, were comparable. Length of hospital stay was significantly prolonged in the cirrhosis group (19 ± 21 vs. 10 ± 4 days, *p* = 0.01).

**Figure 2:**
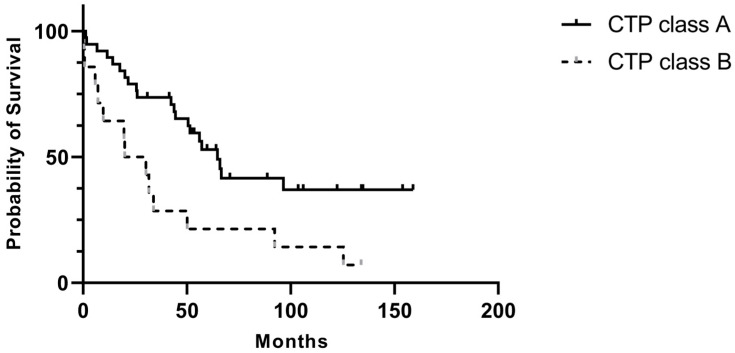
Postoperative overall survival comparing patients with CTP A and CTP B.

Among patients with cirrhosis, 38 had CTP class A, 14 had CTP class B, and one had CTP class C. Patients with CTP class B tended to be older and had lower preoperative hemoglobin and albumin levels than those in class A (Table 3). While the rates of major and minor postoperative complications did not differ significantly between the CTP A and CTP B groups, CTP B patients exhibited higher rates of ICU admission and perioperative blood loss. Tumor size tended to be larger and histologic differentiation poorer in CTP B patients. Furthermore, in this group had significantly shorter survival times (40.1 months vs. 84.8 months, *p* = 0.006) and higher long-term mortality rates (78.6% vs. 36.8%, *p* = 0.04) (Figure 2).

The single patient with CTP class C developed spontaneous bacterial peritonitis and recurrent upper gastrointestinal bleeding due to duodenal ulceration with active oozing during the postoperative period, resulting in a survival duration of only 56 days.

In the analysis of the factors influencing in-hospital mortality, no significant predictors were identified. However, in the analysis of 60-month mortality, a preoperative serum albumin level below 3.5 g/dL was significantly associated with a higher risk of death (HR = 3.93) (Table 4 and Table 5).

## 4. Discussion

Liver cirrhosis is a widespread condition that is associated with high morbidity and mortality. The most common etiologies include alcohol-related liver disease, metabolic-associated fatty liver disease, biliary abnormalities, vascular disorders, and chronic viral hepatitis B or C. Cirrhosis represents the end stage of chronic hepatic inflammation, leading to diffuse fibrosis and architectural distortion of the liver. Progressive fibrosis increases portal vascular resistance, resulting in portal hypertension and circulatory abnormalities characterized by splanchnic arterial vasodilation. Because the splanchnic circulation accounts for approximately one-fourth of systemic vascular resistance, progressive vasodilation leads to reduced effective arterial blood volume, systemic hypotension, and neurohumoral activation. Consequently, sodium and water retention cause plasma volume expansion and ascites formation. Arterial underfilling and vasoconstriction of the renal circulation culminate in hepatorenal syndrome, while increased cardiac output contributes to a hyperdynamic circulatory state and persistent portal hypertension.

Systemic inflammation also plays an important role in the progression of cirrhosis. Arroyo et al. demonstrated that systemic inflammation is closely linked to acute hepatic decompensation [5]. Cirrhosis is also associated with endothelial dysfunction, which may predispose patients to coronary artery disease (CAD). However, the reported incidence and prevalence of CAD in cirrhotic patients are heterogeneous [6,7]. Gu et al. reported that cirrhosis caused by non-alcoholic steatohepatitis or hepatitis C infection increases CAD risk, whereas other etiologies may not [8]. In our cohort, patients with cirrhosis had a higher prevalence of CAD (13.7%) and diabetes mellitus (46.2%). The latter is observed in approximately 30% of cirrhotic patients, primarily due to glucose intolerance and insulin resistance, which worsen as cirrhosis progresses [9]. It accelerates hepatic fibrosis and is an independent predictor of poor prognosis, being associated with ascites, hepatic encephalopathy, and infection [10].

Approximately one-third of patients with liver cirrhosis are managed in the outpatient setting, and among those who are hospitalized, nearly half develop acute kidney injury (AKI) [11]. Approximately 30% of AKI cases arise from intrinsic renal disease, 15–20% from hepatorenal syndrome, and fewer than 1% from postrenal obstruction. Hypoperfusion secondary to hypovolemia accounts for nearly half of all cases [12]. Preventive measures include strict avoidance of nephrotoxic agents, abstinence from alcohol, routine monitoring of renal function and electrolytes, albumin infusion after paracentesis, and antibiotic prophylaxis for spontaneous bacterial peritonitis and variceal bleeding. Renal failure occurs in up to 20% of cirrhotic patients and significantly increases morbidity and mortality. Patients with end-stage renal disease have a defective immune system, which increases their vulnerability for infections and risk of malignancies and atherosclerotic disease [13]. In our study, renal function, reflected by estimated glomerular filtration rate, was lower in cirrhotic patients but did not reach statistical significance, likely due to the limited sample size. Renal impairment is a common concern in patients with liver cirrhosis and contributes substantially to postoperative morbidity and mortality. In addition to renal dysfunction, nutritional status represents another key determinant of surgical outcomes in cirrhotic patients.

Malnutrition affects 40–70% of patients with liver cirrhosis, particularly those with alcohol- or virus-related etiologies. In contrast, cirrhosis secondary to non-alcoholic fatty liver disease or metabolic dysfunction tends to result in malnutrition at a later stage due to reduced oral intake—caused by early satiety, nausea, and altered taste—and impaired nutrient absorption resulting from bile acid deficiency, bacterial overgrowth, and pancreatic insufficiency [14]. Nutritional optimization represents a cornerstone of enhanced recovery after surgery (ERAS) protocols for colorectal surgery, emphasizing preoperative carbohydrate loading and early postoperative feeding to minimize complications, shorten hospital stays, and preserve lean body mass. Nutritional screening using the Royal Free Hospital–Nutritional Prioritizing Tool has demonstrated high sensitivity and specificity in cirrhotic outpatients and correlates strongly with increased risk of hospitalization and mortality [15]. For patients suspected of malnutrition, anthropometric assessments are recommended—specifically focusing on triceps skinfold thickness (below the fifth percentile for age-, sex-, and ethnicity-matched females) to evaluate fat stores and mid-arm muscle circumference (below the fifth percentile for males) to assess muscle mass depletion. Body composition analysis using bioelectrical impedance provides acceptable sensitivity and specificity, along with good reproducibility [14]. In our study, cirrhotic patients exhibited lower preoperative serum albumin levels compared with non-cirrhotic patients (3.3 vs. 3.5 g/dL). Furthermore, hypoalbuminemia (<3.5 g/dL) was independently associated with higher long-term mortality, consistent with previous findings [16].

Anemia is another common comorbidity in cirrhotic patients, affecting up to 75% of this population. The predominant type is normocytic normochromic anemia, resulting from a combination of chronic inflammation, hypersplenism, nutritional deficiencies, bone marrow suppression, and gastrointestinal bleeding. Previous studies have demonstrated a significant inverse relationship between hemoglobin levels and the severity of hepatic decompensation, as reflected by patients’ CTP, MELD, and MELD-Na scores [17]. Furthermore, a low hemoglobin level (≤8.7 g/dL) at discharge has been identified as a predictor of early readmission, contributing to identification of patients who require closer surveillance after discharge [18]. In our cohort, cirrhotic patients exhibited lower preoperative hemoglobin levels and higher MELD-Na scores compared with non-cirrhotic patients, indicating more advanced hepatic dysfunction. The MELD-Na score, in particular, has been recognized as a valuable predictor of postoperative complications. Scores exceeding 10.75 points have been associated with increased risks of surgical site infection, anastomotic leakage, and intra-abdominal complications following colorectal resection [19]. Coakley DO et al. identified MELD-Na as an independent predictor of anastomotic leakage in partial rectal resection and higher scores from 10–14 to 15–19 points increased the overall mortality and overall complications [20]. In our study, the mean MELD-Na score among cirrhotic patients was 12, correlating with greater intraoperative blood loss, longer operative time, higher perioperative transfusion rates, an increase in major complications, higher rates of surgical site infection, and longer hospital stays, consistent with the findings of Han et al. [21].

Perioperative bleeding remains a major concern in cirrhotic patients due to coagulopathy, thrombocytopenia, and portal hypertension. A previous study identified hemorrhage as the most common complication after small bowel surgery in patients with chronic liver disease, although there was no correlation with altered coagulation parameters or the stage of liver disease [22]. Previous studies reported estimated blood losses ranging from 148 to 245 mL and operative durations of approximately 150 min in cirrhotic patients undergoing colorectal resection [21,23]. In our study, the mean blood loss was 166 mL and the mean operative time was 211 min, likely reflecting meticulous hemostasis performed to minimize postoperative hemorrhage. Continuous improvements in surgical techniques and instruments have gradually reduced operative time and blood loss, potentially improving the safety of colorectal surgery for cirrhotic patients.

Postoperative complication rates after colorectal surgery in cirrhotic patients have been reported to range from 36.7% to 77% [21,23,24]. Our overall complication rate (32.1%) was somewhat lower, possibly due to patient selection and the proportion of patients with advanced liver dysfunction. Meunier et al. conducted a study in which 60% patients had CTP class B or C [23] compared to 26.7% of patients in Lee et al.’s study [24]; 16.4% of patients in Han et al.’s study [21]; and 32.1% of patients in our study. The postoperative complication rate increased from 23.2% in CTP class A to 50% in CTP class B, reflecting the influence of hepatic reserve on outcomes.

Specifically, for cirrhotic patients, major possible complications included bleeding, infection, ascites, ileus, and wound healing [24,25,26,27]. Recent reports revealed that postoperative bleeding occurred in approximately 38.9–40.7% of patients, but no patients in our study experienced this [24,28]. During colorectal cancer surgery, we routinely left one intra-abdominal drain. Although ascites or fluid collection occurred in a majority of patients postoperatively, only a few of them required prolonged peritoneal drainage or even additional percutaneous drainage. In our cohort, the wound infection rate was 15.1% in cirrhotic patients, comparable with recent reports (12.3–12.7%), while intra-abdominal abscess occurred in 9.4%, which was higher than the previous report of 1.8%. Interestingly, the severity of liver disease (Child–Pugh A vs. B) did not show a clear correlation with infection rates [24,29]. Liver cirrhosis is always associated with malnutrition and poor wound healing and significantly increases the risk of intra-abdominal abscesses due to impaired immune function and impaired circulation.

Postoperative mortality remains a major concern. In our study, the mean survival was 70.7 months, short-term (3-month) mortality was 9.4%, and long-term (60-month) mortality reached 66%. In Han et al.’s study, the 5-year overall survival in the liver cirrhosis group was significantly lower than that in the non-LC group (46.7% vs. 76.2%, *p* < 0.001). The survival rate could be significantly affected by the poor liver function and mortality associated with liver cirrhosis. Several prognostic tools have been evaluated to predict outcomes in cirrhotic surgical patients. The CTP score is widely used but can be partially subjective, particularly when assessing ascites and hepatic encephalopathy. By contrast, the MELD-Na score, which incorporates serum sodium, provides an objective measure of liver function and predicts postoperative survival more reliably. Prior studies demonstrated that a MELD-Na score < 10 identifies patients as suitable for surgery, whereas higher scores correlate with increased postoperative morbidity and mortality from 54.6% to 27.5% [21]. In our present study series, the postoperative short-term mortality rate was 9.4%, in agreement with previous reports (8.1–11%) [4,30], and the long-term mortality was 66%, which was very similar to findings from previous studies (65%) [31]. In addition, the long-term mortality in patients with CTP class A was 36.8%, while for patients with CTP class B, it was 78.6% with significance, compared to previous findings of 48% and 77%, respectively [31]. The creditable mortality rate was obtained from a meta-analysis within a national database.

Further subgroup analyses revealed that the detrimental effects of cirrhosis persisted across surgical types. Long-term mortality remained significantly elevated in both right hemicolectomy and non-right hemicolectomy groups, with the right hemicolectomy cohort demonstrating particularly poor survival. Although short-term and in-hospital mortality differences did not reach statistical significance, likely due to the small sample size, the numerical trends consistently favored higher mortality in cirrhotic patients.

Interestingly, postoperative outcomes differed by procedure type. Among non-right hemicolectomy patients, cirrhosis was associated with higher ICU admission rates, greater transfusion needs, increased major complications, and prolonged hospitalization. In contrast, cirrhotic patients undergoing right hemicolectomy did not exhibit significantly increased perioperative complications compared with non-cirrhotic patients, suggesting that this procedure may be better tolerated from a physiological standpoint. Nevertheless, the markedly worse long-term survival in the cirrhotic right hemicolectomy group may relate to tumor characteristics typical of right-sided colon cancer, including a more advanced stage at presentation, larger tumor size, and poorer histologic differentiation.

In multivariate analysis, no factors independently predicted short-term mortality, whereas preoperative hypoalbuminemia emerged as the sole independent predictor of long-term mortality. In contrast, univariate analysis identified perioperative transfusion and major complications as significant predictors of short-term mortality. Consistent with previous reports, Meunier et al. found that preoperative peritonitis, postoperative complications, postoperative infections, and total colectomy were associated with increased early mortality (within one month) in cirrhotic patients [27]. Perioperative transfusion likely reflects greater intraoperative blood loss or poorer baseline patient status, while major complications can prolong hospitalization and increase early mortality, even in patients without cirrhosis. Regarding long-term outcomes, malnutrition, as indicated by low preoperative albumin, is a well-established risk factor for reduced survival and increased morbidity in chronic liver disease, independent of surgical insult, and its impact is particularly pronounced following abdominal surgery [28].

Prothrombin time prolongation predicted morbidity in a multivariate analysis of 161 patients with CLD undergoing CRS (15), stressing that a thorough examination of the patient’s hemostatic situation is strongly recommended. Any planned surgical intervention must, therefore, include close consultation with hemostasis experts, and a comprehensive laboratory workup (i.e., repetitive global coagulation tests) seems advisable.

The main limitations of this study include its retrospective design and relatively small sample size, which may limit the statistical power of the findings. Nonetheless, we attempted to minimize selection bias between groups by applying propensity score matching and excluding emergent operations. Despite these limitations, our results provide valuable insights for colorectal surgeons, particularly regarding operative mortality in patients with liver cirrhosis. Preoperative optimization, especially of functional and nutritional status, should be emphasized and further explored in future prospective studies evaluating surgical outcomes in this high-risk population.

## 5. Conclusions

In summary, liver cirrhosis significantly increases postoperative morbidity and mortality in patients undergoing elective colorectal cancer surgery, reducing long-term survival. Right hemicolectomy appears better tolerated, with similar short-term outcomes to those observed in non-cirrhotic patients, though long-term survival remains poorer. Patients with a higher CTP class, elevated MELD-Na scores, and hypoalbuminemia showed worse mortality. Hypoalbuminemia (<3.5 g/dL) was identified as an independent predictor of mortality, underscoring the importance of preoperative nutritional assessment and optimization. Careful perioperative management, risk stratification, and multidisciplinary coordination are essential to improve surgical outcomes in this high-risk population.

## Figures and Tables

**Table 1:** Comparison of clinicopathological characteristics and perioperative outcomes between patients with and without liver cirrhosis and subgroup analysis of patients in right and non-right hemicolectomy groups.

Variable	Cirrhosis (n = 53)	Non-Cirrhosis (n = 53)	*p*-Value
**Age (years, mean ± SD)**	67 ± 10	68 ± 11	0.73
**BMI (kg/m^2^, mean ± SD)**	23.8 ± 3.6	24.8 ± 4.3	0.20
**Gender, male (%)**	71.7	39.6	**0.00**
**Tumor location, n (%)**			
Colon	42 (79.2)	45 (84.9)	0.45
Rectum	11 (20.8)	8 (15.1)	
**Comorbidities, n (%)**			
Coronary artery disease (CAD)	7 (13.7)	1 (1.9)	**0.02**
End-stage renal disease (ESRD)	3 (5.9)	0 (0)	0.07
Diabetes mellitus (DM)	24 (46.2)	13 (24.5)	**0.02**
Heart failure (HF)	5 (9.4)	3 (5.7)	0.46
Stroke	7 (13.2)	6 (11.3)	0.76
Chronic obstructive pulmonary disease (COPD)	1 (1.9)	2 (3.8)	0.56
**Past abdominal operation, n (%)**	1 (1.9)	0 (0)	0.32
**MDRD (mL/min/1.73 m^2^, mean ± SD)**	75.1 ± 29.7	80.3 ± 25.3	0.34
**MELD-Na (mean ± SD)**	12 ± 5	9 ± 3	**0.00**
**Preoperative CEA (ng/mL, mean ± SD)**	16.1 ± 26.9	8.7 ± 25.5	0.15
**Preoperative hemoglobin (g/dL, mean ± SD)**	11.1 ± 2.4	12.1 ± 2.1	**0.03**
**Preoperative CCRT, n (%)**	3 (5.7)	1 (1.9)	0.31
**Preoperative albumin (g/dL, mean ± SD)**	3.3 ± 0.7	3.5 ± 0.7	0.05
**Blood loss (mL, mean ± SD)**	166 ± 251	80 ± 106	**0.02**
**Operation time (min, mean ± SD)**	211 ± 80	180 ± 69	**0.03**
**Perioperative transfusion, n (%)**	11 (20.8)	1 (1.9)	**0.00**
**Postoperative ICU admission, n (%)**	13 (24.5)	2 (3.8)	**0.00**
**Pathological stage, n (%)**			0.91
Stage I	18 (34.0)	17 (32.1)	
Stage II	14 (26.4)	15 (28.3)	
Stage III	16 (30.2)	14 (26.4)	
Stage IV	5 (9.4)	7 (13.2)	
**Tumor size (mm, mean ± SD)**	45.7 ± 25.3	39.6 ± 16.5	0.14
**Histologic differentiation, n (%)**			0.68
Well	3 (5.7)	2 (3.8)	
Moderate	44 (83.0)	47 (88.7)	
Poor	6 (11.3)	4 (7.5)	
**Vascular invasion, n (%)**	5 (9.4)	11 (20.8)	0.10
**Lymphatic invasion, n (%)**	32 (60.4)	39 (73.9)	0.15
**Perineural invasion, n (%)**	15 (28.3)	19 (35.8)	0.41
**Overall complications, n (%)**	17 (32.1)	10 (18.9)	0.095
**Clavien–Dindo classification, n (%)**			
Minor (I–II)	7 (13.2)	8 (15.1)	0.78
Major (≥III)	10 (18.9)	2 (3.8)	**0.01**
**Intra-abdominal abscess, n (%)**	5 (9.4)	0 (0)	**0.02**
**Ileus, n (%)**	6 (11.3)	5 (9.4)	0.75
**Surgical site infection, n (%)**	8 (15.1)	1 (1.9)	**0.02**
**Anastomotic leakage, n (%)**	3 (5.7)	3 (5.7)	1.00
**Chyle leakage, n (%)**	0 (0)	1 (1.9)	0.31
**Length of stay (days, mean ± SD)**	18 ± 17	10 ± 5	**0.00**
**Overall survival (months, mean ± SD)**	70.7 ± 8.6	116.8 ± 8.4	**0.00**
**Mortality, n (%)**			
In-hospital	4 (7.5)	0 (0)	**0.04**
Short-term (≤3 months)	5 (9.4)	0 (0)	**0.02**
Long-term (≤60 months)	35 (66.0)	15 (28.3)	**0.00**
**Right hemicolectomy mortality, n (%)**	**Cirrhosis (n = 21)**	**Non-cirrhosis (n = 17)**	
In-hospital	3 (14.3)	0 (0)	0.10
Short-term (≤3 months)	4(19)	0 (0)	0.06
Long-term (≤60 months)	16 (76.2)	6 (35.3)	**0.01**
**Non-right hemicolectomy mortality, n (%)**	**Cirrhosis (n = 32)**	**Non-cirrhosis (n = 36)**	
In-hospital	1 (3.1)	0 (0)	0.289
Short-term (≤3 months)	1 (3.1)	0 (0)	0.289
Long-term (≤60 months)	19 (59.4)	9 (40.6)	**0.00**

Values are presented as mean ± standard deviation or number (percentage), as appropriate. Abbreviations: BMI—body mass index; CAD—coronary artery disease; COPD—chronic obstructive pulmonary disease; ESRD—end-stage renal disease; DM—diabetes mellitus; MDRD—modification of diet in renal disease; MELD-Na—model for end-stage liver disease sodium score; CEA—carcinoembryonic antigen; CCRT—concurrent chemoradiotherapy; HF—heart failure; ICU—intensive care unit; SSI—surgical site infection; LOS—length of hospital stay. Bold values indicate statistical significance (*p* < 0.05).

**Table 2:** Perioperative and postoperative outcomes between cirrhotic and non-cirrhotic patients in right and non-right hemicolectomy groups.

Variable	Right Hemicolectomy			Non-Right Hemicolectomy		
	Cirrhosis (n = 21)	Non-Cirrhosis (n = 17)	*p*-Value	Cirrhosis (n = 32)	Non-Cirrhosis (n = 36)	*p*-Value
Operation time (min, mean ± SD)	202 ± 81	167± 63	0.16	219 ± 81	187± 42	0.09
Perioperative transfusion, n (%)	4 (19)	0 (0)	0.06	7 (21.9)	1 (2.8)	**0.02**
Postoperative ICU admission, n (%)	6 (28.6)	2 (11.8)	0.206	7 (21.9)	0 (0)	**0.00**
Overall complications, n (%)	8 (38.1)	4 (23.5)	0.34	9 (28.1)	6 (16.7)	0.26
Clavien–Dindo classification, n (%)						
Minor (I–II)	3 (14.3)	3 (17.6)	0.78	4 (12.5)	5 (13.9)	0.87
Major (≥III)	5 (23.8)	1 (5.9)	0.13	5 (15.6)	1 (2.8)	0.06
Intra-abdominal abscess, n (%)	2 (9.5)	0 (0)	0.191	3 (9.4)	0 (0)	0.06
Ileus, n (%)	3 (14.3)	3 (17.6)	0.78	2 (6.3)	3 (8.3)	0.74
Surgical site infection, n (%)	4 (19)	1 (5.9)	0.23	4 (12.5)	0 (0)	**0.03**
Anastomotic leakage, n (%)	2 (9.5)	1 (5.9)	0.68	1 (3.1)	2 (5.6)	0.62
Chyle leakage, n (%)	0 (0)	0 (0)	1	0 (0)	1 (2.8)	0.34
Length of stay (days, mean ± SD)	18 ± 12	12 ± 7	0.06	19 ± 21	10 ± 4	**0.01**

**Table 3:** Comparison of clinical characteristics and outcomes between patients with CTP class A and class B cirrhosis undergoing colorectal cancer surgery.

Variable	CTP A (n = 38)	CTP B (n = 14)	*p* Value
**Age (years, mean ± SD)**	66 ± 10	73 ± 9	**0.025**
**BMI (mean ± SD)**	23.54 ± 3.67	24.78 ± 3.55	0.281
**Gender, Male (%)**	30 (78.9)	7 (50.0)	**0.040**
**Tumor location**			0.179
Colon	29 (76.3)	13 (92.9)	
Rectum	9 (23.7)	1 (7.1)	
**Comorbidities**			
CAD	4 (10.8)	3 (23.1)	0.273
ESRD	3 (7.9)	1 (7.1)	0.909
DM	17 (45.9)	7 (50.0)	0.796
HF	3 (7.9)	2 (14.3)	0.49
Stroke	4 (10.5)	3 (21.4)	0.31
COPD	1 (2.6)	0 (0)	0.54
**Past abdominal operation**	1 (2.6)	0 (0)	0.54
**MDRD (mL/min/1.73 m^2^)**	77.8 ± 31.5	70.6 ± 23.4	0.439
**MELD-Na**	11 ± 4	14 ± 5	0.075
**Preoperative CEA (ng/mL)**	12.9 ± 19.1	25.1 ± 41.7	0.152
**Preoperative Hb (g/dL)**	11.6 ± 2.3	10.0 ± 2.1	**0.027**
**Preoperative CCRT, n (%)**	3 (7.9)	0 (0)	0.279
**Preoperative albumin (g/dL)**	3.6 ± 0.4	2.5 ± 0.6	**<0.001**
**Blood loss (mL)**	116 ± 165	294 ± 286	**0.020**
**Operation time (min)**	217 ± 89	199 ± 52	0.462
**Perioperative transfusion, n (%)**	5 (13.2)	5 (35.7)	0.067
**Postoperative ICU, n (%)**	5 (24.5)	7 (50.0)	**<0.001**
**Pathological stage**			0.050
I	17 (44.7)	1 (7.1)	
II	11 (28.9)	3 (21.4)	
III	9 (23.7)	6 (42.9)	
IV	1 (2.6)	4 (28.6)	
**Tumor size (mm)**	39.1 ± 23.6	63.4 ± 22.6	**0.002**
**Histologic differentiation**			**0.007**
Well	2 (5.2)	1 (7.1)	
Moderate	35 (92.1)	8 (57.1)	
Poor	1 (2.6)	5 (35.7)	
**Vascular invasion, n (%)**	2 (5.3)	2 (14.3)	0.279
**Lymphatic invasion, n (%)**	21 (55.3)	10 (71.4)	0.292
**Perineural invasion, n (%)**	11 (28.9)	3 (21.4)	0.588
**Overall complications, n (%)**	9 (23.7)	8 (51.7)	**0.023**
**Clavien–Dindo classification, n (%)**			
Minor (I–II)	3 (7.9)	4 (28.6)	0.053
Major (≥III)	6 (15.8)	3 (21.4)	0.634
Intra-abdominal abscess	3 (7.9)	2 (14.3)	0.488
Ileus	3 (7.9)	2 (14.3)	0.488
SSI	4 (10.5)	4 (28.6)	0.110
Anastomotic leakage	3 (7.9)	0 (0)	0.279
**LOS (days)**	14 ± 13	28 ± 23	**0.007**
**Survival (months)**	84.8 ± 10.5	40.1 ± 11.6	**0.006**
**Mortality**			
In-hospital	2 (5.3)	1 (7.1)	0.743
Short-term (≤3 months)	2 (5.3)	2 (14.3)	0.254
Long-term (≤60 months)	14 (36.8)	11 (78.6)	**0.040**

Values are presented as mean ± standard deviation or number (percentage), as appropriate. Abbreviations: BMI—body mass index; CAD—coronary artery disease; COPD—Chronic obstructive pulmonary disease; ESRD—end-stage renal disease; DM—diabetes mellitus; MDRD—modification of diet in renal disease; MELD-Na—model for end-stage liver disease sodium score; CEA—carcinoembryonic antigen; CCRT—concurrent chemoradiotherapy; HF—heart failure; ICU—intensive care unit; SSI—surgical site infection; LOS—length of hospital stay.

**Table 4:** Univariate and multivariate analyses of factors associated with in-hospital mortality in patients with liver cirrhosis undergoing colorectal surgery.

Variable	Univariate Analysis (*p* Value)	Multivariate Analysis (*p* Value)
Male	0.990	
Age > 60 years	0.907	
MDRD < 60 mL/min/1.73 m^2^	0.982	
Age > 80 years	0.571	
CTP class B/C	0.332	
Preoperative platelet < 150 × 10^9^/L	0.844	
Preoperative Hb < 10 g/dL	0.228	
Preoperative bilirubin > 1.2 mg/dL	0.285	
Preoperative sodium < 135 mmol/L	0.070	
Preoperative albumin < 3.5 g/dL	0.998	
Preoperative INR > 1.2	0.070	
MELD > 10	0.998	
CAD	0.999	
DM	0.394	
Stroke	0.48	
COPD	1	
HF	0.999	
**Past abdominal operation**	1	
Non-right hemicolectomy operation	0.168	
Postoperative ICU admission	0.241	
**Perioperative transfusion**	**0.025**	0.182
Operative time > 211 min	0.602	
Any complication	0.998	
Minor (Clavien–Dindo I–II)	0.480	
**Major (Clavien–Dindo ≥ III)**	**0.018**	0.128

Abbreviations: CAD—coronary artery disease; CTP—Child–Turcotte–Pugh; COPD—chronic obstructive pulmonary disease; DM—diabetes mellitus; MDRD—Modification of Diet in Renal Disease; Hb—hemoglobin; HF—heart failure; INR—international normalized ratio; MELD—model for end-stage liver disease; ICU—intensive care unit. **Note:** Variables with *p* < 0.05 in univariate analysis were entered into multivariate analysis.

**Table 5:** Univariate and multivariate analyses of factors associated with long-term mortality in patients with liver cirrhosis undergoing colorectal surgery.

Variable	Univariate Analysis (*p* Value)	Multivariate Analysis (*p* Value)	HR
Male	0.561		
Age > 60 years	0.523		
MDRD < 60 mL/min/1.73 m^2^	0.346		
Age > 80 years	0.739		
Child B/C	0.186		
Preoperative platelet < 150 × 10^9^/L	0.930		
Preoperative Hb < 10 g/dL	0.368		
Preoperative bilirubin > 1.2 mg/dL	0.872		
**Preoperative sodium < 135 mmol/L**	**0.043**	0.118	
**Preoperative albumin < 3.5 g/dL**	**0.011**	**0.037**	**3.93**
Preoperative INR > 1.2	0.564		
MELD > 10	0.066		
CAD	0.864		
DM	0.616		
Stroke	**0.999**		
COPD	1		
HF	0.498		
**Past abdominal operation**	1		
Non-right hemicolectomy operation	0.211		
Postoperative ICU admission	0.780		
Perioperative transfusion	0.600		
Operative time > 211 min	0.636		
Any complication	0.202		
Minor (Clavien–Dindo I–II)	0.263		
Major (Clavien–Dindo ≥ III)	0.769		

**Abbreviations:** CAD—coronary artery disease; CTP—Child–Turcotte–Pugh; DM—diabetes mellitus; MDRD—Modification of Diet in Renal Disease; Hb—hemoglobin; INR—international normalized ratio; MELD—model for end-stage liver disease; ICU—intensive care unit. **Note:** Variables with *p* < 0.05 in univariate analysis were included in the multivariate model.

## Data Availability

The datasets analyzed during the current study are not publicly available to protect the privacy of the participants.

## Abbreviations

The following abbreviations are used in this manuscript:

AKI	Acute kidney injury.
BMI	Body mass index.
CAD	Coronary artery disease.
CEA	Carcinoembryonic antigen.
CTP	Child–Turcotte–Pugh.
ERAS	Enhanced Recovery After Surgery.
ESKD	End-stage kidney disease.
ICU	Intensive care unit.
LC	Liver cirrhosis.
MDRD	Modification of Diet in Renal Disease.
MELD-Na	Model for End-Stage Liver Disease-Sodium.
OR	Odds ratio.
SD	Standard deviation.
HR	Hazard ratio.
VOCAL-Penn	Veterans Outcomes and Costs Associated with Liver Disease–Penn Cirrhosis Surgical Risk Score.
